# Judicial Opinion 130

**DOI:** 10.1099/ijsem.0.006414

**Published:** 2024-06-06

**Authors:** David R. Arahal, Carolee T. Bull, Henrik Christensen, Maria Chuvochina, Christopher Dunlap, Maria del Carmen Montero-Calasanz, Charles T. Parker, Peter Vandamme, Antonio Ventosa, Stefano Ventura, Peter Young, Markus Göker

**Affiliations:** 1 Departamento de Microbiología y Ecología, Universitat de València, Valencia, Spain; 2 Department of Plant Pathology and Environmental Microbiology, Pennsylvania State University, 211 Buckhout Lab, University Park, PA 16802, USA; 3 Department of Veterinary and Animal Sciences, University of Copenhagen, Stigbøjlen 4, 1870 Frederiksberg C, Denmark; 4 The University of Queensland, School of Chemistry and Molecular Biosciences, Australian Centre for Ecogenomics, QLD 4072, Australia; 5 Crop Bioprotection Research Unit, USDA/ARS/NCAUR, 1815 N. University St, 61604 Peoria, Illinois, USA; 6 IFAPA Las Torres - Andalusian Institute of Agricultural and Fisheries Research and Training, Cra. Sevilla-Cazalla de la Sierra, 41200, Alcalá del Río, Sevilla, Spain; 7 Department of Energy Joint Genome Institute, Berkeley, CA 94720, USA; 8 BCCM/LMG, Laboratorium voor Microbiologie, Universiteit Gent (UGent) K.L. Ledeganckstraat 35, B-9000 Gent, Belgium; 9 Department of Microbiology and Parasitology, Faculty of Pharmacy, University of Sevilla, Sevilla, C/. Prof. Garcia Gonzalez 2, ES-41012 Sevilla, Spain; 10 IRET-CNR, Research Institute on Terrestrial Ecosystems, National Research Council, Via Madonna del Piano 10, 50019 Sesto Fiorentino, and NBCF, National Biodiversity Future Center, Palermo, Italy; 11 Department of Biology, University of York, York YO10 5DD, UK; 12 Leibniz Institute DSMZ – German Collection of Microorganisms and Cell Cultures, Inhoffenstrasse 7B, D-38124 Braunschweig, Germany

## Abstract

Opinion 130 deals with a Request for an Opinion asking the Judicial Commission to clarify whether the genus name *Rhodococcus* Zopf 1891 (Approved Lists 1980) is illegitimate. The Request is approved and an answer is given. The name *Rhodococcus* Zopf 1891 (Approved Lists 1980) is illegitimate because it is a later homonym of the validly published cyanobacterial name *Rhodococcus* Hansgirg 1884. The Judicial Commission also clarifies that it has the means to resolve such cases by conserving a name over an earlier homonym. It is concluded that the name *Rhodococcus* Zopf 1891 (Approved Lists 1980) is significantly more important than the name *Rhodococcus* Hansgirg 1884 and therefore the former is conserved over the latter. This makes the name *Rhodococcus* Zopf 1891 (Approved Lists 1980) legitimate.

## Introduction

The Request for an Opinion by Goodfellow *et al.* [1] asks the Judicial Commission to clarify whether the genus name *Rhodococcus* Zopf 1891 (Approved Lists 1980) [2,3] is illegitimate. Based on an earlier publication [4], the Request for an Opinion emphasizes that this genus name is a later homonym of *Rhodococcus* Hansgirg 1884 [5,6], a genus of cyanobacteria. Goodfellow *et al.* [1] also note that relatively recent emendations [7,8] of the International Code of Nomenclature of Prokaryotes (ICNP) have the effect that cyanobacterial names validly published under the International Code of Nomenclature for algae, fungi and plants, or ICN [9], are also considered to be validly published under the ICNP [10].

This Judicial Opinion will first clarify whether *Rhodococcus* Zopf 1891 (Approved Lists 1980) [2,3] is illegitimate and what the consequences might be. It then considers whether, if it is illegitimate, the name could be made legitimate and, if so, whether the Judicial Commission should take such a step.

## Is *Rhodococcus* Zopf 1891 (Approved Lists 1980) illegitimate?

*Rhodococcus* Zopf 1891 (Approved Lists 1980) [2,3] is obviously a later homonym of *Rhodococcus* Hansgirg 1884 [5,6]. Rule 51b(4) of the ICNP [10] lists such a case of later homonymy as a possible reason for the illegitimacy of a name. The more recently introduced wording of Rule 18a [3,7, 8] explicitly refers to homonyms of cyanobacterial names. Rule 51a states that a ‘name contrary to a Rule is illegitimate and may not be used’. However, Rules 32b and 54 indicate that epithets are not rendered illegitimate by occurring in combination with an illegitimate genus name. Thus, according to Rule 54 the illegitimacy of *Rhodococcus* Zopf 1891 (Approved Lists 1980) [2,3] could be resolved by proposing new combinations for all species currently placed in the genus for which alternative validly published and legitimate names are not yet available [10]. There are several recently published examples of such replacements [11,18].

As the newly introduced paragraphs of General Consideration 5, Rule 18a and Rule 24a [10] refer to names that are ‘described under the provisions of the International Code of Nomenclature for algae, fungi, and plants’ or (less ambiguously) ‘validly published under the provisions of the International Code of Nomenclature for algae, fungi, and plants’, it is of interest to assess whether *Rhodococcus* Hansgirg 1884 [5,6] is indeed validly published in accordance with the ICN [9].

As exemplified by Articles 37.1, 40.1, 40.4, 40.6, 40.7, 44.1 and 44.2, valid publication under the ICN [9] has become more demanding over time. This includes the ICN’s regulations specific to algae. However, these Articles are non-retroactive, hence *Rhodococcus* Hansgirg 1884 [5,6] is not affected by them. The name was published before the starting points for the nomenclature of ‘*Nostocaceae homocysteae*’ and ‘*Nostocaceae heterocysteae*’ specified in Article 13.1 [9], but since *Rhodococcus* Hansgirg 1884 [5,6] belongs to the non-filamentous cyanobacteria, these starting points do not apply to it. *Rhodococcus* Hansgirg 1884 was proposed harbouring a single species, *Rhodococcus caldariorum* Hansgirg 1884, which became the type species by monotypy according to Articles 40.3 and 40.6 [9].

In the same year, both Hansgirg [5] and Wittrock and Nordstedt [6] published Hansgirg’s description of *Rhodococcus* and *Rhodococcus caldariorum*. While Hansgirg [5] mentioned that *Rhodococcus caldariorum* would later be reported by Wittrock and Nordstedt (“Wird in Wittrock’s und Nordstedt’s ‘Algae aquae dulcis exsiccatae’ mitgetheilt werden”), he gave Latin descriptions of both genus and species on pp. 314–315. There is no indication that Hansgirg did not accept the genus *Rhodococcus* at this time; the term ‘vorläufig’ (provisionally) on p. 315 refers to the inclusion of this genus, along with *Chroococcus* Nägeli 1849 and *Pleurococcus* Meneghini 1837, in an unnamed taxon apparently above genus rank (but in a category called ‘Reihe’, i.e. series). In addition, on p. 316, Hansgirg expresses the view that it would be inappropriate to treat *Rhodococcus* together with the other two genera as a subgenus of the common genus (‘Sammelgattung’) *Coccus*.

Therefore, Hansgirg’s own descriptions [5] meet the requirements of Articles 33.1 and 36.1 [9]. Wittrock and Nordstedt [6] gave different Latin descriptions on p. 128, apparently based on the same material, and attributed them to Hansgirg. It is not clear to the Judicial Commission which of the two papers was published earlier and therefore it is not clear which of the two publications constitutes the effective publication of the names of the genus and its type species. However, the Judicial Commission has no doubt that they are validly published under the ICN [9] and that the year of valid publication is 1884.

For these reasons, the Judicial Commission agrees with the authors of the Request for an Opinion [1] that *Rhodococcus* Zopf 1891 (Approved Lists 1980) [2,3] is illegitimate because it is a later homonym of *Rhodococcus* Hansgirg 1884 [5,6]. This conclusion was put to the vote; 12 Commissioners voted in favour, none voted against and none abstained. The remainder of this Judicial Opinion is therefore based on that decision.

## Could *Rhodococcus* Zopf 1891 (Approved Lists 1980) be made legitimate?

The Request for an Opinion [1] does not mention that the Judicial Commission may have the means to resolve such cases of illegitimacy, namely by conserving the later name over the earlier name [10,19]. The first question to be addressed is whether this possibility extends to the resolution of cases of illegitimacy, or whether it can only resolve cases of unpleasant priority [19]. The second question to be addressed is whether conservation by the Judicial Commission can include names validly published under the ICN [9].

The wording of Rule 23a Note 4(1) and Rule 56b indicates that conservation extends to homonyms, which in turn is linked to Rule 51b [4,10]. Therefore conservation of a name or epithet is a means of solving an instance of ‘a later homonym of a name of a taxon of prokaryotes, fungi, algae, protozoa or viruses’ because a conserved name ‘must be used instead of all earlier … homonyms’ [10]. Furthermore, Rule 18a(3) states that in ‘cases of homonymy, wherein the name of a cyanobacterial taxon was published under both codes, the oldest name has priority’ and it would be illogical to treat cases of homonymy between a cyanobacterial name and a non-cyanobacterial name differently. However, while a later homonym may not by default be the correct name of a taxon under Rule 23a, the ‘Judicial Commission may make exceptions to Rule 23a by the addition of names to the list of conserved names (nomina conservanda)’ as indicated in Rule 23a Note 1 [10].

Apart from that, there are two precedents which clarify both questions. In Judicial Opinion 9 [20], the Judicial Commission conserved the name *Gallionella* Ehrenberg 1838 (Approved Lists 1980) [3,21] over an earlier homonym used in botany, *Gaillonella* Bory 1823; the difference in spelling being considered a better latinisation. Judicial Opinion 9 noted that ‘The name *Gallionella* as a genus of diatoms has quite disappeared in the literature of algology’ and expected the rejection of this diatom name by the relevant committee on botanical nomenclature. However, the Judicial Commission has so far found no evidence that this rejection has taken place.

Judicial Opinion 12 [20] conserved the bacterial name *Listeria* Pirie 1940 (Approved Lists 1980) [3,22] over the name of a flowering plant, *Listeria* Necker 1790, that was not even considered for rejection by a committee on botanical nomenclature. Judicial Opinion 12 noted that ‘the suppression of … *Listeria* as bacterial generic names would cause much confusion and resentment. The generic name *Listeria* Necker … has not been used by botanists since the time of its introduction (over a period of more than a century and a half)’.

The purpose of the two Judicial Opinions was, of course, to revoke the illegitimacy of the two names. Otherwise these actions would have been meaningless. The wording of Judicial Opinions 9 and 12 also indicates that these actions established the legitimacy of the names in question, which were considered to be later homonyms. Moreover, both Judicial Opinions based the conservation of the respective name on its greater practical relevance compared to the earlier synonym. The question is therefore whether *Rhodococcus* Zopf 1891 (Approved Lists 1980) [2,3] is of significantly greater practical relevance than *Rhodococcus* Hansgirg 1884 [5,6].

## Should *Rhodococcus* Zopf 1891 (Approved Lists 1980) be made legitimate?

Concerning *Rhodococcus* Hansgirg 1884, the author, Anton Hansgirg, proposed 1 year later [23] on p. 382 to transfer the type species, *Rhodococcus caldariorum* Hansgirg 1884, to the genus *Chroococcus* Nägeli 1849 [24] as *Chroococcus caldariorum* (Hansgirg 1884) Hansgirg 1885. Hansgirg also gave a description of *Chroococcus caldariorum* in German on p. 159 of his 1892 work [25], in which he treated *Rhodococcus* as a section of *Chroococcus*. As *Chroococcus caldariorum* appears to be validly published and legitimate under the ICN [9], a name for the type species of the genus *Rhodococcus* Hansgirg 1884 would remain available even if the name of the genus was made unavailable by conserving *Rhodococcus* Zopf 1891 (Approved Lists 1980) over it.

This would be true even if there were no earlier heterotypic synonyms of *Rhodococcus caldariorum* Hansgirg 1884 ≡ *Chroococcus caldariorum* (Hansgirg 1884) Hansgirg 1885. However, Komárek and Anagnostidis [26] treat this species on p. 243 as a later heterotypic synonym of *Gloeocapsa bituminosa* (Bory 1823) Kützing 1849 [27] ≡ *Chroococcus bituminosus* (Bory 1823) Hansgirg 1892 [25] (pp. 224 and 165, respectively).

No other species within *Rhodococcus* Hansgirg 1884 are known to the Judicial Commission. The names *Rhodococcus* Hirose 1958 and *Rhodococcus caldarius* (Tilden 1898) Hirose 1958 [28], introduced for a red alga, are illegitimate under the ICN because *Rhodococcus* Hirose 1958 is also a later homonym of *Rhodococcus* Hansgirg 1884.

*Rhodococcus* Zopf 1891 (Approved Lists 1980) [2,3] is a much larger genus [29] containing dozens of species with names validly published under the ICNP [10]. Some species are relevant in applied areas such as bioremediation [30,31] and sustainable agriculture [32,33] while others pose a risk to human, animal or plant health [34,35]. The genus includes species that are therefore assigned to risk group 2 [36]: *Rhodococcus aichiensis* corrig. Tsukamura 1983, *Rhodococcus chubuensis* Tsukamura 1983 and *Rhodococcus obuensis* Tsukamura 1983 [37,38] as well as *Rhodococcus gordoniae* Jones *et al.* 2004 [39,40]. No validly published synonyms placed in another genus are available for the latter three species names.

In contrast, *Rhodococcus equi* (Magnusson 1923) Goodfellow and Alderson 1977 (Approved Lists 1980) [3,41] has been proposed to be placed in the genus *Prescottella* as *Prescottella equi* (Magnusson 1923) Sangal *et al.* 2022 [42,43], along with few other species. *R. equi* is of importance as a pathogen of foals and of immunocompromised humans [34,44, 45]. Its name was addressed in Judicial Opinion 106, which placed the epithet *hoagii* in its putative heterotypic synonym *Rhodococcus hoagii* (Morse 1912) Kämpfer *et al.* 2014 [46,47] ≡ *Corynebacterium hoagii* (Morse 1912) Eberson 1918 (Approved Lists 1980) [3,48] on the list of rejected epithets [49].

*Rhodococcus fascians* (Tilford 1936) Goodfellow 1984 [50,51] causes fasciation in plants [34,35]. Together with some other *Rhodococcus* species, it has been proposed to be placed in the genus *Rhodococcoides* Val-Calvo and Vázquez-Boland 2023 [52,53]. However, the majority of species names in *Rhodococcus* Zopf 1891 (Approved Lists 1980) lack a synonym validly published and legitimate under the ICNP [29]. It also remains to be seen how well the *Rhodococcoides* or *Prescottella* names will be appreciated by practitioners.

## Conclusion

The negative consequences of the current illegitimacy of *Rhodococcus* Zopf 1891 (Approved Lists 1980) [2,3] are therefore greater than those of a possible future illegitimacy of *Rhodococcus* Hansgirg 1884 [5,6]. As long as *Rhodococcus* Zopf 1891 (Approved Lists 1980) is illegitimate, this name and the names of the species in this genus are not available for use under the ICNP and would need to be replaced [10]. For this reason, the Judicial Commission decided to conserve *Rhodococcus* Zopf 1891 (Approved Lists 1980) over *Rhodococcus* Hansgirg 1884. Twelve Commissioners voted in favour of this move, none voted against and none abstained. The Judicial Commission would like to point out that the same kind of action can, in principle, be applied to any other case of inconvenient homonymy.

## Addendum

While voting on this Opinion, the Judicial Commission was saddened to learn of the passing of Professor Michael Goodfellow. A world-renowned expert on actinobacteria (*Actinomycetota*), Professor Goodfellow established numerous national and international collaborations and taught countless students during his decades-long career. Professor Goodfellow served the Judicial Commission twice: in Class 15 from 1978 to 1986 and in Class 19 from 1994 to 2002. He will be greatly missed.

## Abbreviations

ICN: International Code of Nomenclature for algae, fungi and plant
ICNP: International Code of Nomenclature of Prokaryotes
